# Engineering safe anti-CD19-CD28ζ CAR T cells with CD8a hinge domain in serum-free media for adoptive immunotherapy

**DOI:** 10.3389/fimmu.2025.1545549

**Published:** 2025-05-09

**Authors:** Muthuganesh Muthuvel, Thamizhselvi Ganapathy, Trent Spencer, Sunil S. Raikar, Saravanabhavan Thangavel, Alok Srivastava, Sunil Martin

**Affiliations:** ^1^ Laboratory of Synthetic Immunology, Cancer Research Division, Biotechnology Research Innovation Council- Rajiv Gandhi Centre for Biotechnology (BRIC - RGCB), Department of Biotechnology, Thiruvananthapuram, India; ^2^ Manipal Academy of Higher Education (MAHE), Manipal, Karnataka, India; ^3^ Center for Stem Cell Research (CSCR), Christian Medical College (CMC) Vellore, Velllore, India; ^4^ Cell and Gene Therapy Program, Aflac Cancer and Blood Disorders Center, Department of Pediatrics, Children’s Healthcare of Atlanta and Emory University School of Medicine, Atlanta, GA, United States; ^5^ Department of Hematology, Christian Medical College (CMC) Vellore, Velllore, India; ^6^ Haematology Research Unit, St. John’s Research Institute, St. John’s National Academy of Health Sciences, Bengaluru, Karnataka, India

**Keywords:** CD19 CAR T cells, B lineage malignancies, serum free media, SIN vector, NALM-6

## Abstract

**Background:**

Despite the curative potential, high cost of manufacturing and the toxicities limits the wider access of Chimeric Antigen Receptor (CAR) T cell therapy in global medicine. CARs are modular synthetic antigen receptors integrating the single-chain variable fragment (scFv) of an immunoglobulin molecule to the TCR signaling. CARs allow HLA independent, T cell mediated destruction of tumor cells independent of tumor associated-HLA downregulation and survive within the patient as ‘living drug.’ Here we report a safer approach for engineering alpha beta T cells with anti- CD19-CD28ζ CAR using self-inactivating (SIN) lentiviral vectors for adoptive immunotherapy.

**Method:**

αβ T cells from the peripheral blood (PB) were lentivirally transduced with CAR construct containing hinge domain from CD8α, transmembrane and co-stimulatory domain from CD28 along with signaling domain from CD3ζ and driven by human UBC promoter. The cells were pre-stimulated through CD3/CD28 beads before lentiviral transduction. Transduction efficiency, fold expansion and phenotype were monitored for the CAR T cells expanded for 10–12 days. The antigen-specific tumor-killing capacity of CD19 CAR T cells was assessed against a standard CD19 expressing NALM6 cell lines with a flow cytometry-based assay optimized in the lab.

**Results and conclusion:**

We have generated high titer lentiviral vectors of CAR with a titer of 9.85 ± 2.2×10^7^ TU/ml (mean ± SEM; n=9) generating a transduction efficiency of 27.57 ± 2.4%. (n=7) at an MOI of 10 in total T cells. The product got higher CD8+ to CD4+ CAR T cell ratio with preponderance of an effector memory phenotype on day 07 and day 12. The CAR-T cells expanded (148.4 ± 29 fold; n=7) in serum free media with very high viability (87.8 ± 2.2%; n=7) on day 12. The antitumor functions of CD19 CAR T cells as gauged against percentage lysis of NALM6 cells at a 1:1 ratio is 27.68 ± 6.87% drawing up to the release criteria. CAR T cells produced IFNγ (11.23 ± 1.5%; n=6) and degranulation marker CD107α (34.82 ± 2.08%; n=5) in an antigen-specific manner. Furthermore, the sequences of WPRE, GFP, and P2A were removed from the CAR construct to enhance safety. These CAR T cells expanded up to 21.7 ± 5.53 fold with 82.7±5.43% viability (n=4).

**Conclusion:**

We have generated, validated, and characterized a reproducible indigenous workflow for generating anti-CD19 CAR T cells *in vitro.* This approach can be used for targeting cancer and autoimmune diseases in which CD19+ B lineage cells cause host damage.

## Introduction

B lineage lymphoid malignancies have been the target of early CAR T cell development for creating newer options for those relapsing after initial therapy for these otherwise highly curable diseases. Despite impressive research in the academia and industries, lack of access and availability to the advanced cell therapies such as CAR cell therapy is a major challenge and remains as an unmet healthcare problem especially in resource limited settings across the world (1).

Cytokine release syndrome (CRS) and the consequent immune effector cell associated neurotoxicity (ICANs), immune effector cell-associated hematotoxicity (ICAHT), and other off-target effects are also some of the limitations of CAR T cell therapies. Although effective, CD28 based CARs tends to generate more inflammatory lymphokines and resulted in ICANS in at least 50% patients with stage 3 and above CRS which translates to more hospitalization cost apart from multiorgan damages (2). Together with other investigators, it has been demonstrated that alteration in the receptor configuration at the level of hinge and transmembrane domain remains to be one of the most effective approaches to resolve the challenges of CAR therapy in the clinics. Kochendefer’s group reported a significant reduction in toxicity without compromising efficacy when the hinge and transmembrane domain is altered with CD8α instead of CD28 (3, 4). An optimized receptor design yielding a highly efficient, less toxic and an affordable CAR therapy is yet to be achieved.

The protocols established for CAR T cells therapy in the clinical trials are tuned for high yield, antitumor potential, and stable expression of CAR transgene. The process flow of cell-based therapy is cautiously evolving to incorporate and purge accessory reagents and alternative approaches. Conventionally, CAR T cells are generated by viral transduction of the primary T cells from the peripheral blood of the patient with an insert coding for CAR receptor followed by expansion in media containing homeostatic cytokines such as IL-2, IL-15, and IL-7. Serum free/Xeno free media with known chemical composition is being increasingly used to mass produce safe products with reduced batch-to-batch variation (5). A safe viral vector and transduction protocol for reducing insertional mutagenesis apart from other unintended toxicities are critical to improve the long-term benefits of CAR therapy. Self-inactivating vector (SIN) vectors are considered as an industry standard across lentiviral vector systems (6). Importantly the choice promoter that drives the CAR express determines the level of receptor on the surface. While strong promoters with synthetic or xenogeneic origin drives high-level of expression of the transgene and consequent promoter methylation, can result in gene silencing. Although the promoter choice depends on several factors, human origin promoters such as hUBC had been demonstrated to drive stable transgene expression with minimal methylation (7, 8). As surface expression of receptor recognize the target antigen, it is important to understand the cellular dynamics and time kinetics of CAR expression in an ongoing immune response. Protein targets, reporter genes and secondary antibodies that target scFvs were increasingly being used to detect the dynamic surface expression of chimeric antigen receptors (9). Of the late, universal antibodies targeting the linker region of the scFv, namely G4S were used to monitor the surface expression. Such reagents also help in enriching and purifying CAR T cells.

To make safe and medically appropriate CAR therapy available to all the tertiary care hospitals, decentralized academic manufacturing in ambulatory settings is suggested. Although multiple CAR T cell manufacturing workflows with reduced cost of production are established, increased demand and reduced supply is projected to be a challenge across public healthcare soon (10). Furthermore, repurposing of CD19 CAR T cells to treat non-malignant disorders such as autoimmune diseases can further increase the demand for product.

In this context, we have indigenously optimized cGMP compatible workflow for enhanced safety with T cells expanded in chemically defined media, SIN vector with CAR transgene driven by human UBC promoter. The reduced multiplicity of infection and receptor design to diminish the chance of insertional mutagenesis. Additionally, altered receptor design with hinge derived from CD8α as opposed to CD28 ensure reduced cytokine toxicity without compromising cytokine production and antigen specific tumor lysis capacity.

## Materials and methods

### Cell lines

CD19 expressing NALM6 cell line were purchased from ATCC and cultured in RPMI media with 10% FBS and 1% Penstrep as per ATCC recommendations. CD19 knockout NALM6 cell line were generated at our campus at RGCB using CRISPR/Cas9 approach. HEK 293T purchased from ATCC was used as a producer cell line for lentiviral particles.

### Lentiviral production and titration

The CAR construct employed is a second-generation CAR with a single-chain variable fragment (scFv) from the FMC63 antibody (Spencer and Raikar Laboratory at Emory University, Atlanta, USA). The scFv is linked to the cytoplasmic domain of CD28 and CD3ζ, through the CD8α hinge and CD28 transmembrane domain. The complete insert is linked to eGFP by a ribosome skipping domain-P2A. A CAR construct without the scFv is used as control in certain experiments. For generating 3^rd^ generation pseudo-lentiviral particles, HEK 293T cells were transfected with transfer plasmid (CAR), packaging plasmids (pRSV-Rev and pMDLg/pRRE) and envelope (pMD2.G) using calcium phosphate method. Pseudovirus were concentrated from the supernatants by ultracentrifugation (26000 rpm for 2 hours) and titered via GFP expression of HEK 293T cells transduced with the virus at various dilutions (1/10,1/100,1/1000 etc.). Dilutions with less than 10% GFP expression were used to calculate the viral titer expressed as transduction unit (TU) per ml using the formula TU/mL = (number of cells transduced x percent fluorescence)/(virus volume in mL) (**Supplementary Figure 1**).

### Expansion of anti-CD19 CAR-T cells

The study was approved by CSCR/CMC (IRB#11135) and RGCB human ethical committee (lHECl112022_1/01) to perform experiments on human T cell. Peripheral blood was collected from healthy volunteers with informed consent and T cells from the blood were isolated using a T cell negative selection kit (Stem Cell Technologies). Isolated T cells were then stimulated with CD3/CD28 beads (1:1) (Thermo Fisher Scientific) in the presence of IL-2 (100 IU/ml, Miltenyi Biotech) in the serum-free media (Lymphocyte Growth Medium-3, Lonza). After 48 hours of stimulation, the cells were transduced with CAR lentiviral vector and polybrene (8μg/ml, Sigma-Aldrich) via spinfection. Cells were de-beaded on day +3 with replacement of fresh media. The phenotype of the CAR-T cells was measured on day +7 and day +12.

### Phenotyping and flow cytometry

Transduction efficiency of CAR was assessed by GFP expression and CD19-PE (ACROBiosystems). CAR-T cells were characterized phenotypically with anti-human antibodies of CD3, CD4, CD8, CD27, CD45RA, and PD-1 by flow cytometry. The antibodies were mixed in FACS buffer (PBS+2%FBS) and added to the cells. After 30 minutes of incubation, cells were washed twice in FACS buffer at 1000rpm for 5 minutes. 7-AAD was used to stain the dead cells. All antibodies and 7-AAD were purchased from BD Biosciences. The cells were then acquired by BD FACS Celesta and were gated on 7-AAD negative and CD3 positive population and analyzed by *Flowjo 9.1* software.

### *In vitro* cytotoxicity of CD19 CAR-T cells

A flow-based cytotoxicity was modified to measure the cytotoxicity of CAR T cells. Briefly, the untransduced T cells and CAR T cells were co-cultured with CD19(+) NALM6 and CD19(-) cell lines at 1:1 effector to target ratios in a U bottom 96 well plate for 4 hours. The plates were incubated at 37°C with 5% CO2 for 4 hours. The cells were stained with anti-CD3 antibody and 7-AAD to exclude the dead cells form the live population. The GFP and CD3 double negative populations were enumerated to calculate the survival of the tumor cells. The percentage survival of the target cells was calculated by:


$$\%\;Survival=\left[\%CD3^{neg}GFP^{neg}\left(Tumor\;x\;CAR\;T\;cells\right)\;-\;\%CD3^{neg}GFP^{neg}\left(Tumor\;alone\right)\right]$$

% Lysis was calculated by: % specific lysis = 100% survival

### Degranulation and intracellular cytokine analysis

CAR T cells with scFv and CAR T cells without scFv were co-cultured with NALM6 cells at 1:1 ratio in 48 well plate with CD107a and protein transport inhibitor (BD GolgiStop™) and incubated at 37°C with 5% CO2 for 5 hours. Cells were fixed and intracellularly stained with anti-IFNγ antibody as per the manufacturer’s protocol (BD fixation and permeabilization kit). BD FACS Aria III used for acquisition and data were analyzed using FlowJo 9.1.

### Western blot analysis

The primary CAR-T cells and untransduced T cells were collected and washed twice with cold 1x PBS. The cell lysate was prepared by adding RIPA lysis buffer (Sigma-Aldrich) with 1x protease inhibitor cocktail to the pellet. The BCA method used to measure the protein concentration (Pierce™ BCA Protein Assay Kit, Thermo). 25μg of protein lysate was prepared in 1X sample loading buffer and loaded in 12% SDS PAGE. After resolving, proteins were transferred to the PVDF membrane and blocked with 5% bovine serum albumin for 1 hour at room temperature. The blots were stained with primary (1:1000) antibodies overnight in the shaker at 4°C. Following primary antibodies are used: Mouse anti-human CD3ζ (Biolegend), and rabbit anti-human β-actin (Cell Signalling Technology) were used. HRP-conjugated goat anti-rabbit IgG (Cell Signalling Technology) or goat anti-mouse IgG (Cell Signalling Technology) was used as a secondary antibody (1:2000), and blots were developed with Clarity ECL reagent (Bio-Rad) on iBright Imaging Systems (Thermo Fisher Scientific).

### CAR construct

The CAR construct used for most of the experiments is an FMC63 based second generation CAR with hinge derived from CD8 and transmembrane and co-stimulatory domains derived from CD28 followed by the signaling domain – CD3ζ. The CAR transgene is bicistronic linked to eGFP by a ribosome skipping P2A sequence. For the generation of cGMP construct, P2A, GFP and WPRE sequences were removed from the CAR construct by molecular cloning. The constructs were confirmed by sanger sequencing and restriction digestion.

### Statistical analysis

Experiments were performed in biological and technical replicates. GraphPad Prism 9.1 used to analyze the data and generate the graph. Error bars are represented as mean ± SEM. P-value was measured by Two-tailed unpaired t-tests. Significance was indicated by *p<0.5, **P<0.01, ***p<0.001, ****p<0.0001.

## Results

### Generation and expansion dynamics of CD19 CAR-T cells

**Figure 1:**
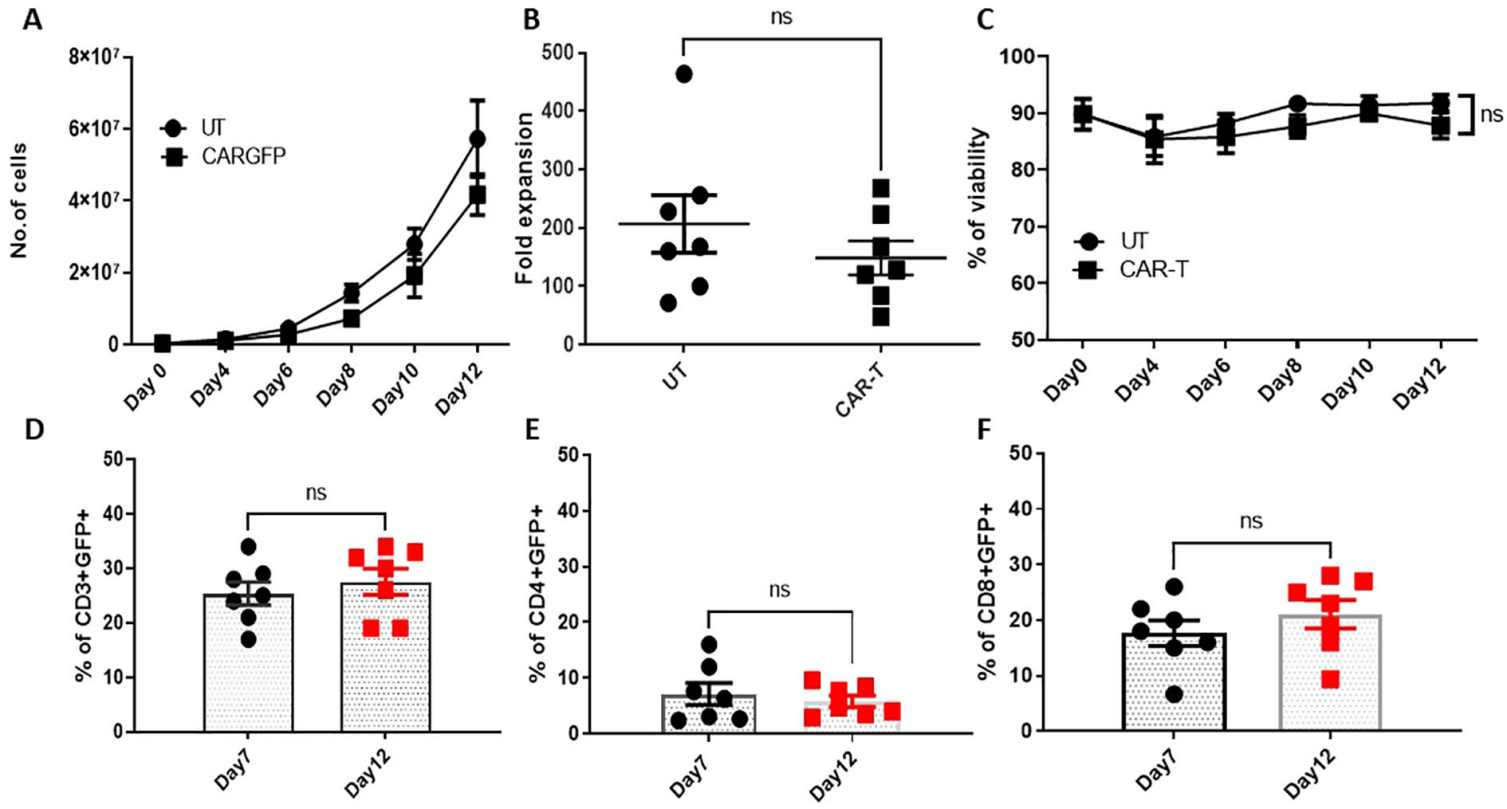
Expansion dynamics of CAR-T cells. **(A)** Graph representing the number of untransduced T cells and CAR transduced T cells at the indicated days after initiating the T cell expansion culture. **(B)** Fold expansion of the untransduced and CAR-transduced T cells on day 12. **(C)** Viability of the untransduced and CAR-transduced cells in the expansion culture. **(D–F)** T cells were transduced with CAR LV vectors, and GFP expression was monitored on day 7 and day 12 as a surrogate of CAR expression in CD3+ **(D)**, CD4+ **(E)**, and CD8+ **(F)** compartments. The data represented as mean ± SEM from seven individual donors (n=7). The statistical significance is estimated by the Student’s T-test. ns, not significant.

Our strategy for generating CAR T cells involves lentiviral transduction of CAR constructs, which were pre-stimulated and expanded for 12 days, for characterization. CD3+T cells were stimulated with CD3**/**CD28 beads (1:1) in the presence of rhIL-2 (100IU) in serum-free media (LGM3, Lonza). High titer lentiviral constructs were generated and tittered, T- cells were transduced with CAR containing scFv and CAR devoid of scFv (*Control*) lentiviral vectors using polybrene (8µg/ml) via spinoculation. The lentiviral vector used for CAR T cell generation is self-inactivating with a mutation in the 3’LTR of the viral genome. Cells were de-beaded on day 03 with replacement media and rhIL-2 followed by monitoring GFP or hCD19-PE expression as a measure of transduction efficiency. Cells were expanded for 12 days with IL-7 (10ng/mL) supplementation for the last 4 days of the culture (**Supplementary Figure 2**). The fold expansion of CAR T cells was found to be 148.4 ± 29 fold; n=7 with 87.8± 2.2% viability, which is comparable to the expansion of untransduced T cells (**Figures 1A–C**). The transduction efficacy of the CAR on day 12 as measured by GFP was found to be 27.57 ± 2.4%, 5.84 ± 1.0 and 21.05 ± 2.5% in the CD3+, CD4+ and CD8+ compartments respectively. On day 07, CD3+GFP+ cells in the CAR transduced set were found to be 25.42 ± 4.2%, whereas by day 12 it increased to 27.57 ± 2.4% (**Figure 1D**). The percentage of CD4+CAR+ cells were observed to be low while CD8+CAR+ cells contributed to 80% of the CAR T cell product (**Figures 1E, F**). The transduction was on par with the cGMP release criteria which is >20% (11). As the surface expressed CAR binds to its cognate antigen, we measured the expression levels on the cell surface. We observed that at least 50% of the CARs are expressed on the cell surface as evidenced by the binding of PE labelled CD19 protein (**Supplementary Figure 3**) and total expression by western blotting against anti-CD3ζ antibody (**Supplementary Figure 4**).

### Memory phenotyping CAR-T cells

T cells are heterogeneous populations with its subsets playing unique role in cancer immunosurveillance. Hence, we tested the markers of memory differentiation as it has been reported that memory cells positively co-relate with the durable antitumor immune response. Using CD45RA and CD27 as markers, we segregated total T cells to naïve (CD45RA^pos^CD27^neg^), central memory cells (CD45RA^neg^CD27^pos^), effector memory cells (CD45RA^neg^CD27^neg^) and (CD45RA^pos^CD27^pos^) effector memory RA cells (EMRA). Our results show that the memory differentiation pattern is similar across treatments including that of the CAR positive population. We also observed an increase in the EMRA population followed by naïve populations. However, there is a decreasing trend in the central and effector memory subsets as it progressed to day 14 (**Supplementary Figure 5**). It is to be noted that the PD-1 expression in the untransduced and CAR T transduced sets remained the same (**Supplementary Figure 6**).

### Antigen specific proliferation of CD19 CAR T cells

**Figure 2:**
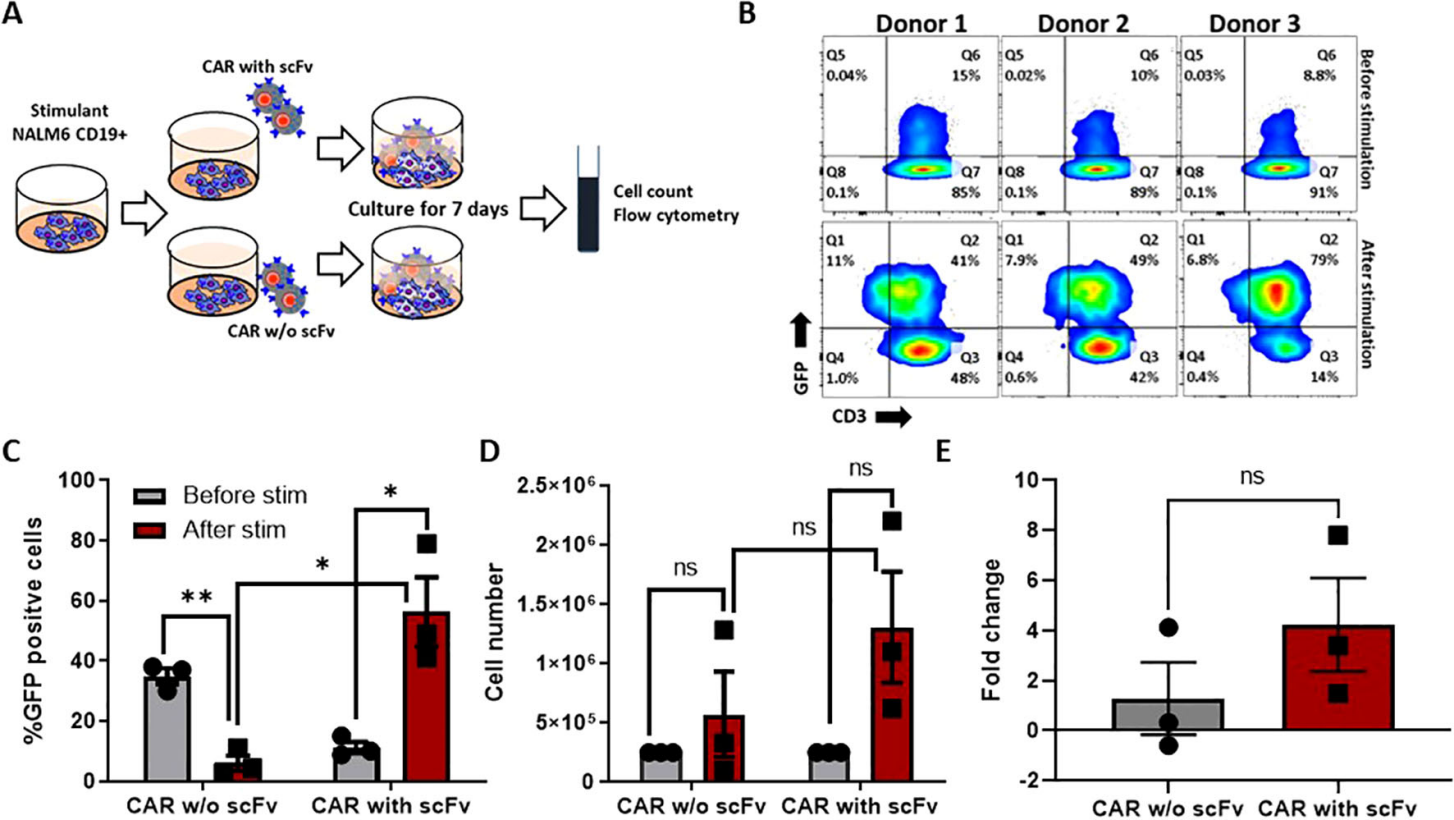
Antigen-specific stimulation of CAR-T cells. **(A)** Experimental design of antigen-specific expansion, Nalm-6 cells were co-cultured with CAR-T cells and CAR without extracellular domain (scFv) at 1:1 ratio and expanded for 7 days. **(B)** Expression of CAR was measured before and after the 7 days of co-culture by flowcytometry. **(C, D)** The CAR expression **(C)** and cell number **(D)** differences were measured before and after stimulation. **(E)** The fold change of antigen-specific proliferation for CAR-T cells and CAR without extracellular domain. The data represented as mean ± SEM from three individual donors (n=3). The statistical significance is estimated by using Student’s T-test. * p<0.05, **p<0.005, ns, not significant.

Upon encounter with the antigen positive tumor cells, CAR T cells proliferate in the patient’s system. Indeed, this was observed when CD19 CAR T cells expressing CD19 binding scFv were co-cultured against CD19 positive and negative NALM6 cells. In comparison to the scFv (-) GFP (+) CAR T cells, we observed an enrichment of scFv (+) GFP (+) CAR T cells after 07 days of co-culture (CAR without scFv 6.06 ± 2.5; CAR with scFv 56.33 ± 11, n=3 Mean ± SEM). CAR T cells co-cultured with the CD19 expressing NALM6 cells significantly expanded in two out of the three donors (Total number of cells: without scFv 5.3X10^5 ± 3.8; with scFv 4.6X10^6 ± 4.6; n=3 Mean ± SEM). We observed a 3.7-fold increase in the antigen specific expansion of the CAR T cells compared to the control, suggesting the premise that CD19 CAR T cells generated can proliferate and potentially persist in patients (**Figures 2A–E**).

### Antitumor functions of CD19 CAR T cells

**Figure 3:**
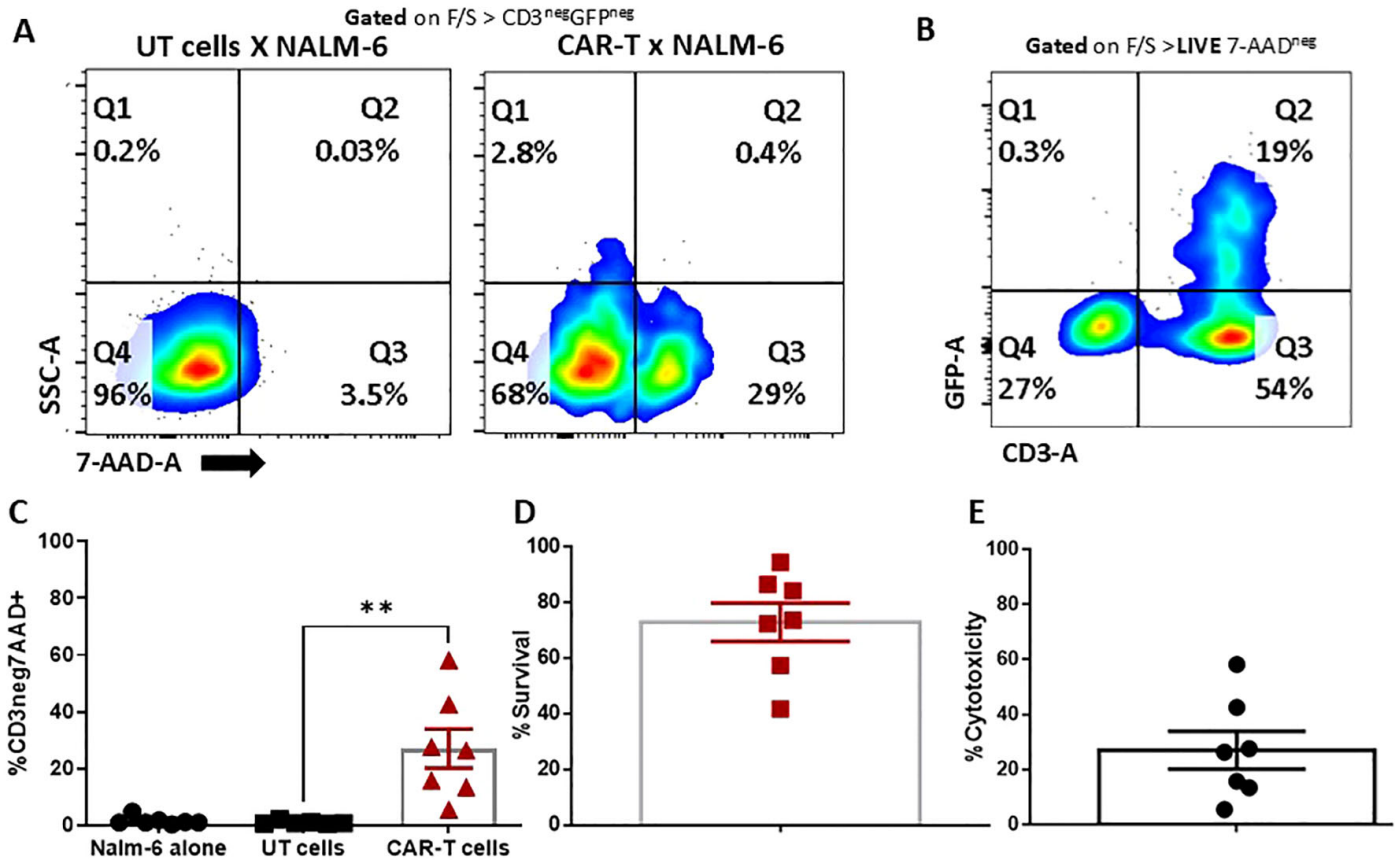
Cytotoxicity of CAR T cells. Nalm-6 cells co-cultured with UT and CAR-T cells at a 1:1 ratio. **(A)** Cells were gated from CD3+ and GFP+ to exclude the CAR-T cells, and from the negative SSC and 7-AAD to measure the lysis of Nalm-6 cells. **(B, C)** Cells were gated from 7-AAD negative and CAR-T cell populations, which were excluded by CD3 and GFP staining. The negative population is the survival of target cells. **(D, E)** Lysis (%CD3neg7AAD+) **(D)** and survival percentage **(E)** of target cells (n=7). The data represented as mean ± SEM from seven individual donors (n=7). The statistical significance is estimated by using Student’s T-test. **p<0.005.

The primary function of CD19 CAR T cells is its ability to cause antigen-specific immunogenic cytolysis of the tumor cells. We employed a flow cytometry-based cytotoxicity assay to gauge the tumor lysis capacity of CAR T cells *in vitro*. In this assay, CD19(+) and CD19(-) NALM6 were the target cells, with the CAR T cells being the effector. NALM6 cells were co-cultured with GFP co-expressing CD19 CAR T cells for 4 hours and the 7-AAD staining (necrotic and dead cells) was measured in the surviving CD3(-) and GFP (-) population. We observed that across 7 donors, the percentage cytotoxicity at 1:1 ratio is 27.68 ± 6.87% and the percentage survival is 72.92 ± 6.87%, which is approaching the release criteria. The transduction efficiency was indicated by the GFP positive population (**Figure 3**). Percentage cytotoxicity was much significant compared to the untransduced T cell co-cultured with CD19+ NALM6 cells (**Supplementary Figures 7**, **8**). We confirmed the compliance of our CAR T cells with the release criteria prescribed for the industry (~30% at 1:1 effector to target ratio for a 4 hours assay) (11). The CAR T cell lyse the CD19(+) NALM6 cells 12 hours and 24 hours co-culture effector to target ratios of 05:01 and 10:01 respectively (**Supplementary Figure 9**).

**Figure 4:**
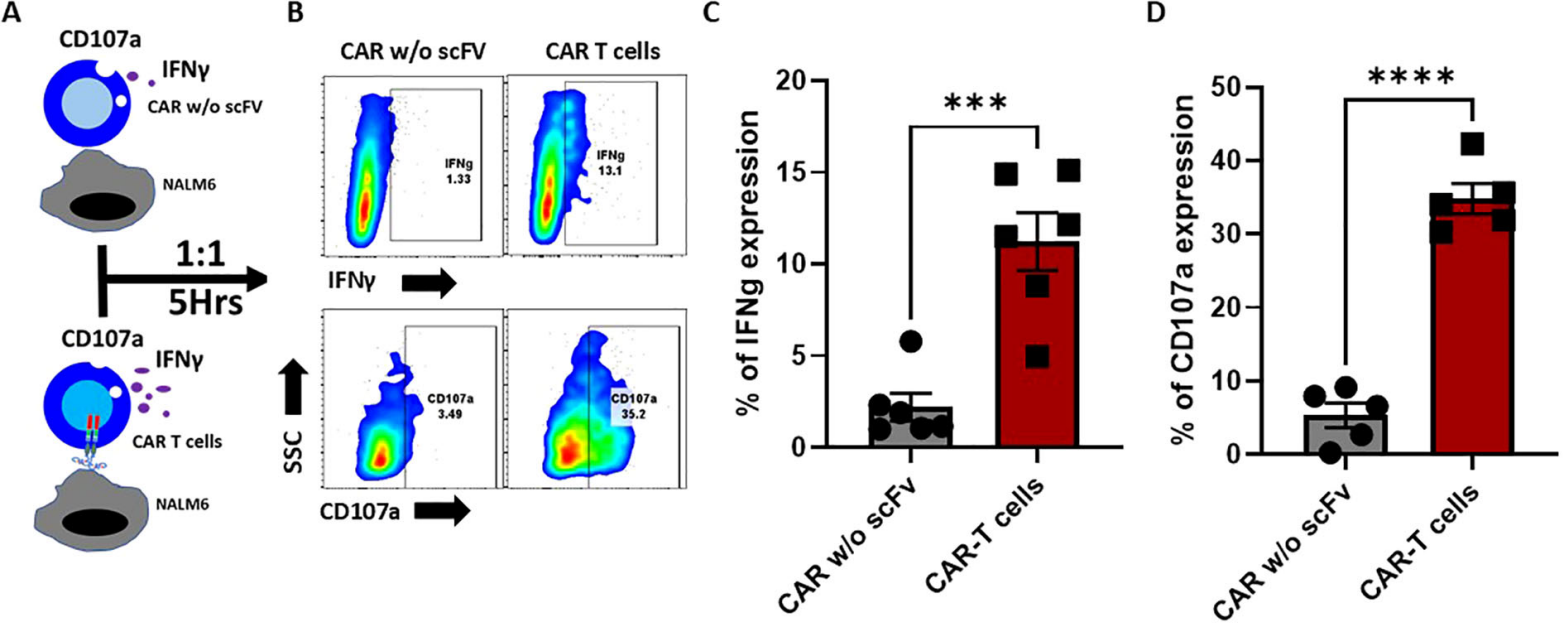
Antigen-specific IFNγ secretion and CD107 degranulation of CD19 CAR-T cells. **(A)** Schematic representation of antigen-specific IFNγ secretion and CD107 degranulation of CD19 CAR-T cells and mock CAR-T cells (without extracellular domain). Effector cells were co-cultured with an equal number of target cells for 5 hours. **(B)** FACS plots representing the percentage of intracellular IFN-γ, and the degranulation was assessed by measuring the surface expression of CD107a. **(C, D)** Bar graph representing the Interferon-gamma **(C)** percentage and CD107a **(D)**. The data are represented as mean ± SEM from six individual donors (n=6) for Interferon-gamma and five individual donors (n=5) for CD107a. The statistical significance is estimated by using the Student’s T-test. ***p<0.001, ***p<0.0001.

The antigen specific anti-tumor functions of CAR T cells are typically measured against the production of IFNγ and CD107a degranulation assays. Therefore, using the NALM6 - CD19 CAR T cell co-culture set up for 5 hours, we monitored the release of IFNγ. T cells expressing CAR without scFv cells was used as an additional control. Results show that CD19 CAR T cells, but not the CAR T cells without scFv, releases the IFNγ and degranulated with surface expression of CD107a upon CD19 recognition, indicating the antigen-specific effector functions of the CAR T cells that was generated (**Figure 4**).

NSG™ is an excellent murine model to study the anti-tumor functions of human immune system against established human xenografts. Therefore, we tested the tumor lysis capacity of CD19 CAR T cells in an NSG™ model of B-ALL established by grafting human NALM-6 cell line. We observed a significant reduction in the tumor burden in a pilot animal trial (n=2) *(data not shown).*

### Generation of CAR constructs without WPRE, GFP and P2A

**Figure 5:**
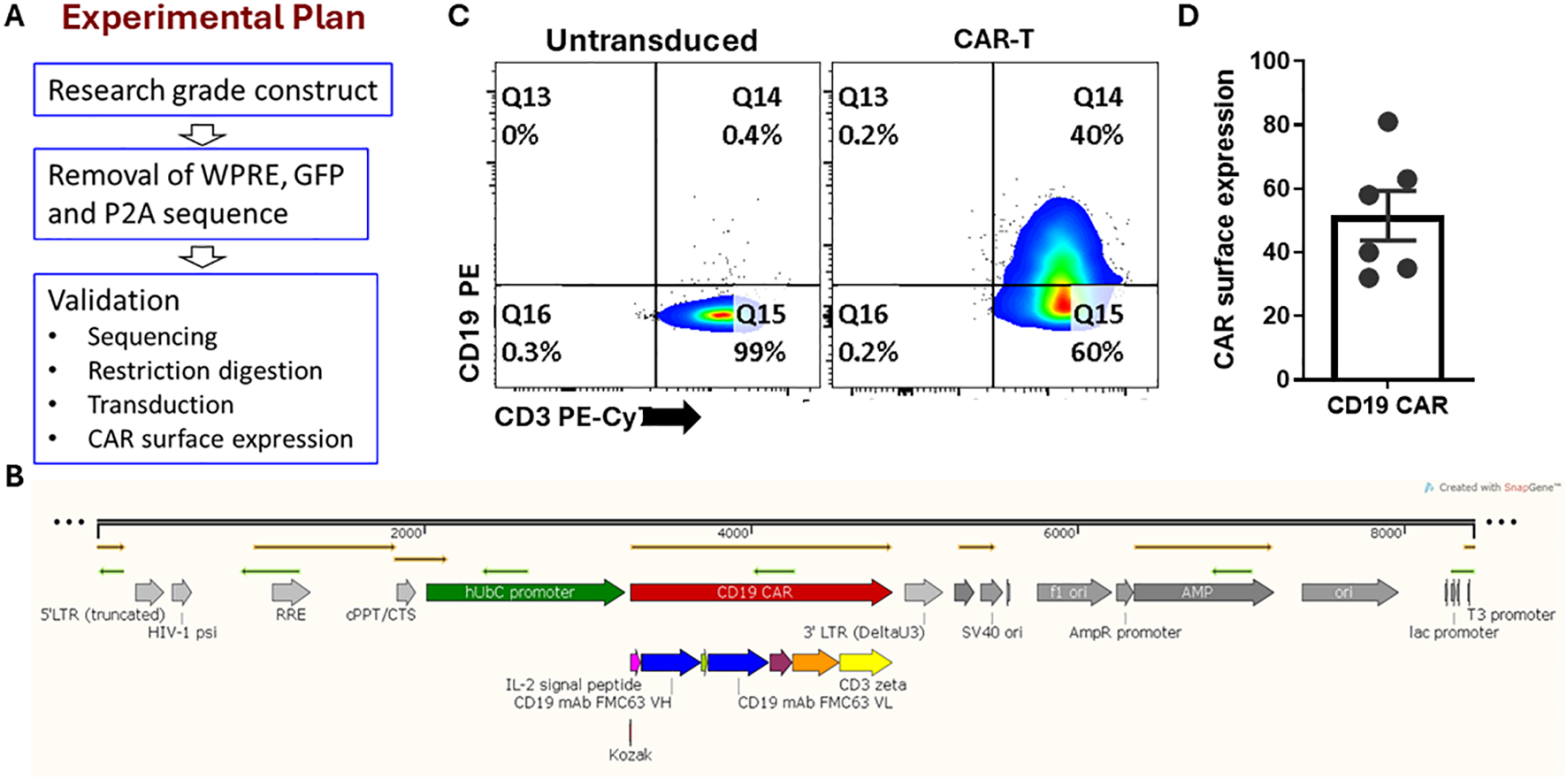
Construction of CD19 CAR construct (without WPRE/GFP/P2A). **(A)** Experimental plan for generating and validating the cGMP grade CAR construct. **(B)** A map of the modified cGMP grade CAR constructs. **(C, D)** The surface expression of CAR was measured by CD19-PE staining across the six different donors (n=6). Data is represented as mean ± SEM.

Generating a cGMP grade CAR construct is essential for future clinical applications. As genetic elements such as GFP, WPRE and P2A is not ideal in cGMP CAR construct, we deleted them, which not only reduced the size of the plasmid but also enhanced the compliance of these constructs with cGMP guidelines (12). The plasmid was then validated by restriction digestion and sequencing. Further, we lentivirally transduced the modified CAR construct and assessed the surface expression of the receptor by staining with CD19 protein bound to PE. The surface expression of CAR was found to be 51.5 ± 7.8% (**Figure 5**). Most of the CD3+ cells had higher CD45RA+CD27+ naïve cells and CD45RA+CD27+ effector memory RA+ cells. Both central and effector memory RA+ cells were minor components (**Supplementary Figures 10D, E**). However, compared to the untransduced cells, the CAR transduced T cells got only very low proliferation rate (**Supplementary Figures 10A, B**). In summary, we have optimized a protocol to lentivirally transduce αβ T cells with CD19 CAR and test its tumor lysis functions *in vitro*. This work may be validated for cGMP grade expansion of CAR T cells.

## Discussion

Here, we have optimized an indigenous protocol for serum-free expansion of CD19 CAR T cells using self-inactivating lentiviral vectors which are the industry standard in safety (13, 14). Moreover, we restricted the multiplicity of infection of transduction to 10 to reduce the insertion of multiple copies of transgene per cell and the consequent insertional mutagenesis. Moreover, a promoter derived from human UBC (hUBC) ensured reduced gene silencing, which was otherwise a major challenge for the stable long-term expression of CAR transgene (15). Although a high-titer virus could be developed, batch to batch variations in transduction efficiency of the pseudo-viral preparation prevented it; however, this can be resolved by producing the virus in a single batch.

Yeskarta and Breyanzi are the two CAR T cell formulations using CD28 co-stimulatory domains. It has been demonstrated that the CRS and the consequent ICANs in these formulations were significantly reduced by exchanging the transmembrane domain and hinge region with CD8α (16). In view of reducing the toxicity without compromising efficacy, our CAR design incorporates hinge domain from CD8α and transmembrane domain from the CD28 region (3). We also generated sequence confirmed CAR constructs devoid of WPRE, P2A and GFP with higher transduction efficiency and memory phenotype profile.

Chemically defined media with xeno-free components is being increasingly used for the expansion of therapeutic immune cells (17). To maintain batch to batch consistency, we used LGM3™ media (Lonza) that was specially formulated to support lymphocyte growth and devoid of exogenous growth factors and artificial stimulators or undefined supplements. Most of the components used for the expansion such as CD3/CD28 beads, IL-2, polybrene and LGM3™ media are cGMP compatible.

CAR T cells generated in our lab proliferated to 150-fold and 87% viability, which is approaching industry standard. The antigen specific expansion of CAR T cells when co-cultured against CD19(+) but not CD19(-) tumor cells indicates the higher on-tumor effect of the CAR T cells. Moreover, this platform would permit us to study the tumor-specific molecular determinants of CAR T cell proliferation. This T cell expansion protocol is enriching the effector memory RA+ population which translates to senescent/exhausted cells. The increased expansion of CD8 compared to CD4 CAR T cells may indicate reduced persistence (18). In the CAR T cell subset, there is an enrichment of effector memory population akin to CAR T cells with CD28 co-stimulatory domain (19). The lower percentage of central memory T cell populations can be a limitation of this protocol. We have modified the flow cytometry-based cytotoxicity assay in which the cells are stained only after the assay (20). This assay will also help us to concurrently monitor the cell death of both T cells and tumor cells. Importantly, there exists a discrepancy between IFNγ and CD107a expression. Not all CAR T cells that generates IFNγ is lysing the tumor cells. Hence, it is important to check the percentage of IFNγ positive and IFNγ negative CAR T cells. The percentage of cytotoxicity is equivalent to the percentage of CAR transduction. Therefore, improving the percentage expression by CAR mRNA preparation can enhance cytotoxicity (ongoing work). Selecting the T cell subsets such as stem cell - like memory cells combined with alteration in the receptor configuration is yet another approach (21).

Overall, the CAR T cells which we developed is an indigenous workflow for expanding anti CD19 CAR T cells with hinge region derived from CD8α and transmembrane and co-stimulatory domain derived from CD28 with signaling domain from CD3ζ. We employed a very safe lentiviral SIN vector with hUBC promoter. Our serum free expansion of CAR T cells has viability, antigen-specific proliferation, tumor killing capacity and cytokine production. We also indigenously generated a cGMP-compatible CAR vector, which is ready for further development as a potential clinical product.

## Data Availability

The original contributions presented in the study are included in the article/**Supplementary Material**. Further inquiries can be directed to the corresponding author.

## Supplementary material

Supplementary Figure 1 Generation of CAR lentiviral particles. **(A)** Diagram of CAR-GFP construct. **(B)** Schematic representation of lentiviral production. **(C)** The titer of the CAR viral particle is based on GFP expression in transduced HEK293T cells, and it is expressed as titration unit (TU)/ml. The data is represented as mean ± SEM (n=9).

Supplementary Figure 2 Diagrammatic workflow for CAR-T cell generation. T cells were isolated directly from peripheral blood or PBMC using T cell negative selection kit (EasySep™ Human T Cell Isolation Kit). The cells were plated at a density of a million per ml of media with recombinant human IL-2 (100IU/mL) (Lymphocyte growth media, Lonza) and stimulated with CD3CD28 beads at a 1:1 ratio. After 48 hours of stimulation, cells were transduced with lentiviral vectors in the presence of polybrene. The cells were debeaded on day 3, and the transduction efficiency was measured on day 7. The cells were expanded in IL-7-containing media during the last 4 days to rest the T cells. Functional assays were carried out on days 12 to 14.

Supplementary Figure 3 Surface expression of CAR. **(A)** The surface expression of CAR was measured in CAR with and without scFv cells using CD19 protein conjugated with PE. **(B)** FACS plot and **(C)** bar graph representing the CAR expression on day 7 and day 12 from three different donors (n=3).

Supplementary Figure 4 Western blot analysis of CAR protein expression in primary T cells. The cell lysate was prepared from the primary T cells transduced with CAR and untransduced T cells from three different donors. The protein lysate was subjected to SDS-PAGE (12%) and immunoblotting with an **(A)** anti-CD3ζ antibody. β-Actin **(B)** was used as a loading control. The expected size of CAR is ~56 kDa. The protein ladder is mentioned on the left side of the blot.

Supplementary Figure 5 Memory phenotype of CAR-T cells. CAR-T cells’ memory phenotype was measured using CD45RA and CD27 staining. **(A)** FACS plots representing untransduced and CAR transduced T cells for day 0, day 7 and day 12. **(B, C)** The graph represents the percentage expression of effector memory (CD45RA^−^CD27^−^), central memory (CD45RA^−^CD27^+^), naïve (CD45RA^+^CD27^+^), and RA^+^ effector memory (CD45RA^+^CD27^−^) subsets in untransduced (B) and CAR-transduced **(C)** T cells, measured across five different expansions (n=5). **(D, E)** Representative pie chart showing only day 12 of untransduced **(D)** and CAR transduced cell **(E)** expansions. Data is represented as mean ± SEM.

Supplementary Figure 6 PD-1 expression during CAR-T cell expansion. **(A)** Representing FACS plot of PD-1 expression measured on days 0, 7, and 12 during CAR-T cell expansion using a flow cytometer. **(B)** Data are represented as a bar graph of the percentage of PD-1 on untransduced T cells, and CAR-T. The data is represented as mean ± SEM (n=3). The statistical significance was estimated by using the Student’s T-test. There were no significant differences between UT and CAR-T cells during expansion.

Supplementary Figure 7 Schematic representative of cytotoxicity assay. The Target(T) NALM-6 cells (CD19 + and CD19 -) were plated at 10^5^ cells/well in a U bottom 96 cell plate in a serum-free media. The effector (E) cells (CAR-transduced cells) and untransduced controls were plated against the targets at a 1:1 (effector-to-target) ratio for 4 hours. The cells were stained with 7-AAD and CD3 antibody and acquired using flow cytometry. They were then serially gated on FSC/SSC gates to exclude the debris and 7-AAD to exclude the dead cells. GFP- CD3- cells were gated for measuring the survival of the target cells.

Supplementary Figure 8 Cytotoxicity of CD19 CAR against an equal number of WT and CD19KO NALM-6 cells. **(A)** Gating strategy to assess the cytotoxicity of CAR T cells against CD19 positive and CD19 negative Nalm-6 cells. The first gate excludes the debris, followed by gating at 7-AAD negative live population. GFP+ and CD3- cells are monitored to assess the survival of Nalm-6 cells. **(B)** FACS plots represent the survival of CD19-positive Nalm-6 cells when co-cultured with CAR T cells at a 1:1 ratio in triplicates. **(C)** FACS plots represent the survival of the CD19-negative Nalm-6 cells. The upper row represents cytotoxic cell lysis by 7-AAD staining alone. **(D, E)** The percentage of 7AAD **(D)** and survival of tumor cells **(E)** are depicted as mean ± SEM (r=3). The statistical significance is estimated by using the Student’s T-test. ****p<0.0001.

Supplementary Figure 9 Cytotoxicity of CAR-T cells at various E:T ratios and time points. CAR-T cells and untransduced T cells (effector) were co-cultured with nalm-6 cells (target) at different effector-to-target ratios (1:1, 5:1, and 10:1) at multiple time points (4 **(A)**, 12 **(B)**, and 24 hours **(C)**). Target cell lysis was measured using a flow cytometry-based cytotoxicity assay. The percentage of cytotoxicity is plotted against various E:T ratios. Data represent mean ± SEM from three different donors (n=3). The statistical significance was estimated by using the Student’s T-test. *p < 0.05, and **p<0.005.

Supplementary Figure 10 Proliferation and differentiation of T cells transduced with cGMP grade CAR constructs. **(A)** The graph represents the number of untransduced and CAR-transduced T cells at the indicated days after initiating the T cell expansion culture. **(B)** Fold expansion of the untransduced and CAR-transduced T cells on day 12. **(C)** Viability of the untransduced and CAR-transduced T cells in the expansion culture. **(D)** Naïve (CD45RA+CD27+), T-cell effector RA positive (CD45RA+, CD27-), central memory (CD45RA-CD27+), effector memory (CD45-CD27-) profile of the CAR T cells on day 12. **(E)** The bar graph shows only day 12 of untransduced T cell and CAR-transduced T cell expansions. Data is represented as mean ± SEM (n=4). The statistical significance is estimated by using the Student’s T-test. ***p<0.0001, ns: not significant.
