# Supplementary figures and images for: Engineering safe anti-CD19-CD28ζ CAR T cells with CD8a hinge domain in serum-free media for adoptive immunotherapy
Source: Front Immunol. 2025 May 9;16:1545549. doi: 10.3389/fimmu.2025.1545549 (PMC12098533; doi:10.3389/fimmu.2025.1545549)

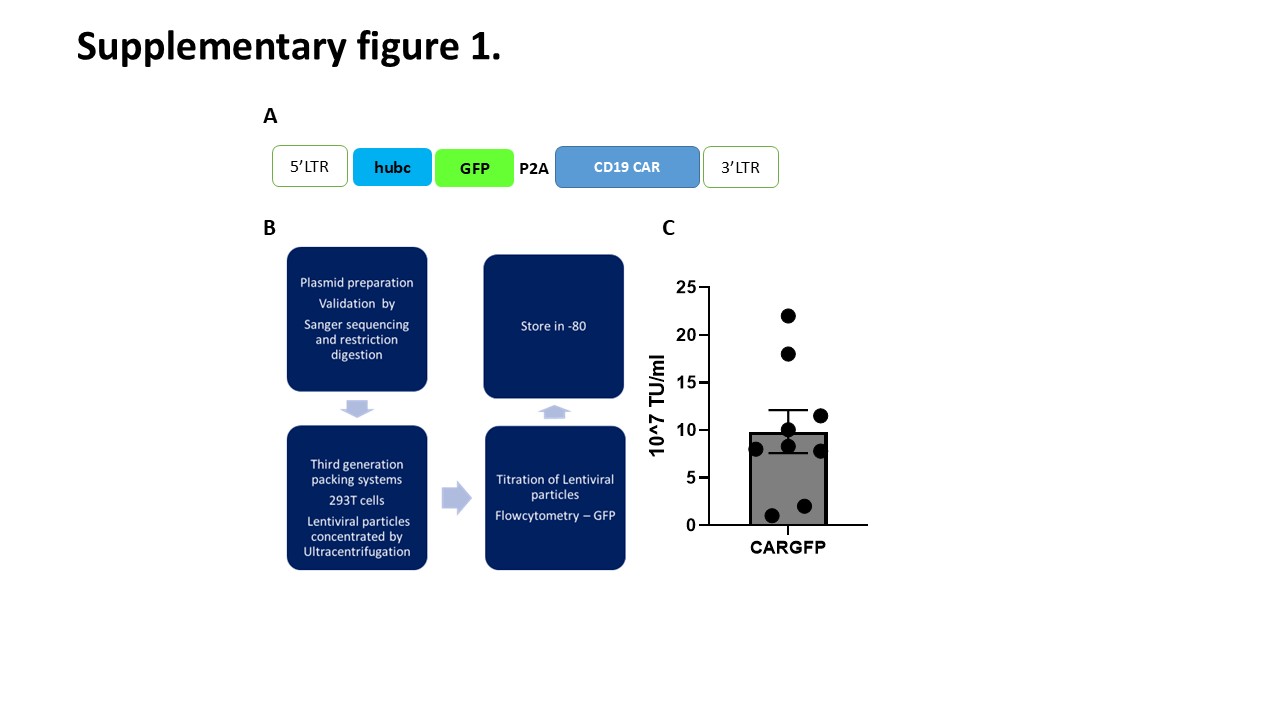

Supplement: Supplementary Figure 1 — Generation of CAR lentiviral particles. (A) Diagram of CAR-GFP construct. (B) Schematic representation of lentiviral production. (C) The titer of the CAR viral particle is based on GFP expression in transduced HEK293T cells, and it is expressed as titration unit (TU)/ml. The data is represented as mean ± SEM (n=9). [file Image1.jpeg]

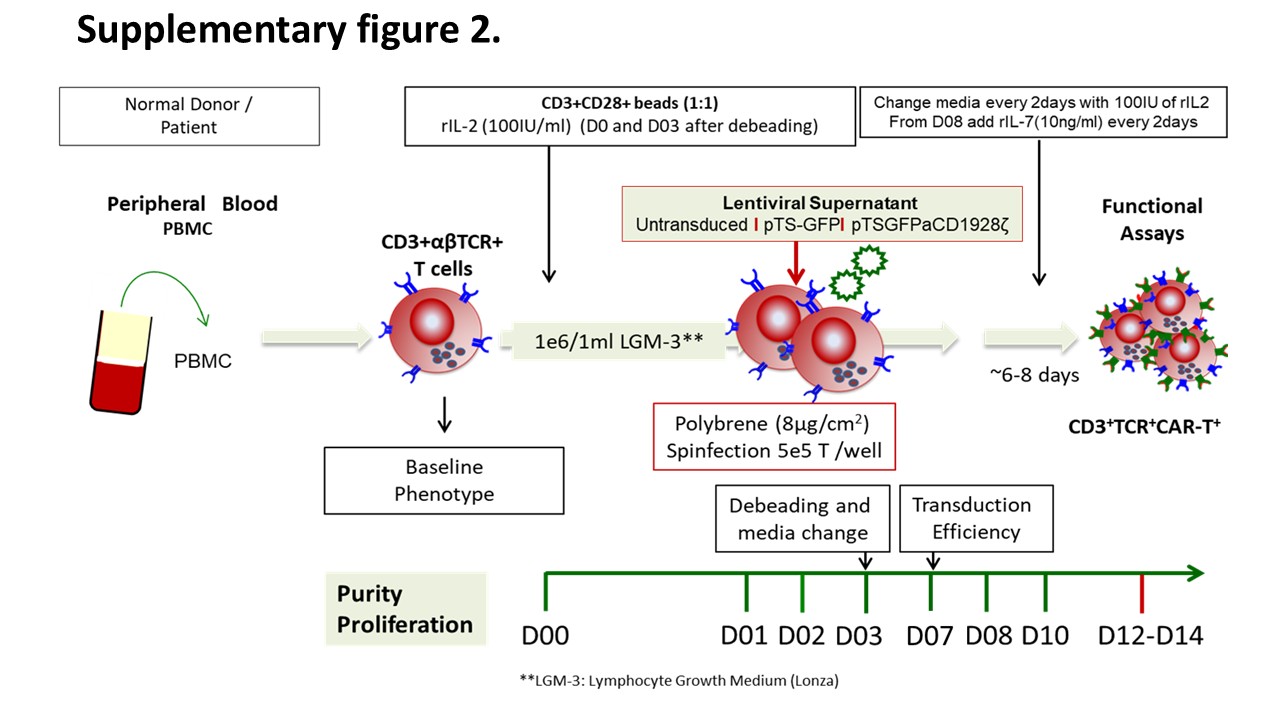

Supplement: Supplementary Figure 2 — Diagrammatic workflow for CAR-T cell generation. T cells were isolated directly from peripheral blood or PBMC using T cell negative selection kit (EasySep™ Human T Cell Isolation Kit). The cells were plated at a density of a million per ml of media with recombinant human IL-2 (100IU/mL) (Lymphocyte growth media, Lonza) and stimulated with CD3CD28 beads at a 1:1 ratio. After 48 hours of stimulation, cells were transduced with lentiviral vectors in the presence of polybrene. The cells were debeaded on day 3, and the transduction efficiency was measured on day 7. The cells were expanded in IL-7-containing media during the last 4 days to rest the T cells. Functional assays were carried out on days 12 to 14. [file Image2.jpeg]

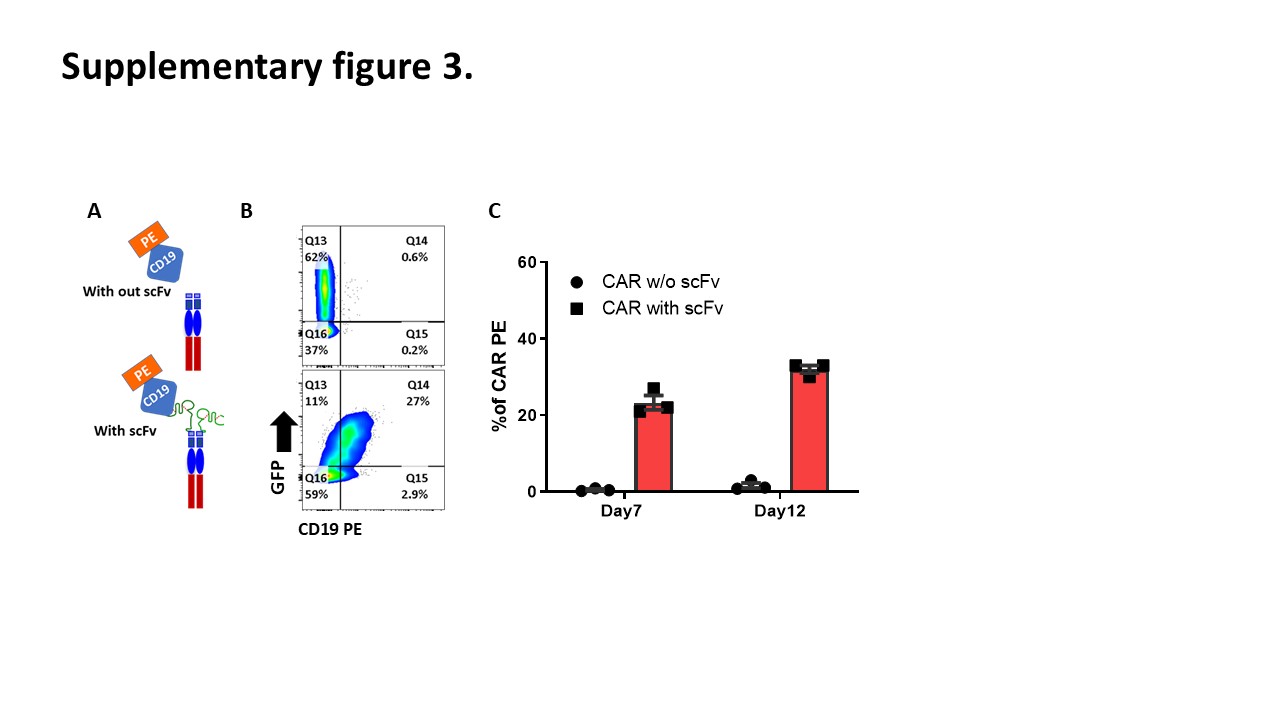

Supplement: Supplementary Figure 3 — Surface expression of CAR. (A) The surface expression of CAR was measured in CAR with and without scFv cells using CD19 protein conjugated with PE. (B) FACS plot and (C) bar graph representing the CAR expression on day 7 and day 12 from three different donors (n=3). [file Image3.jpeg]

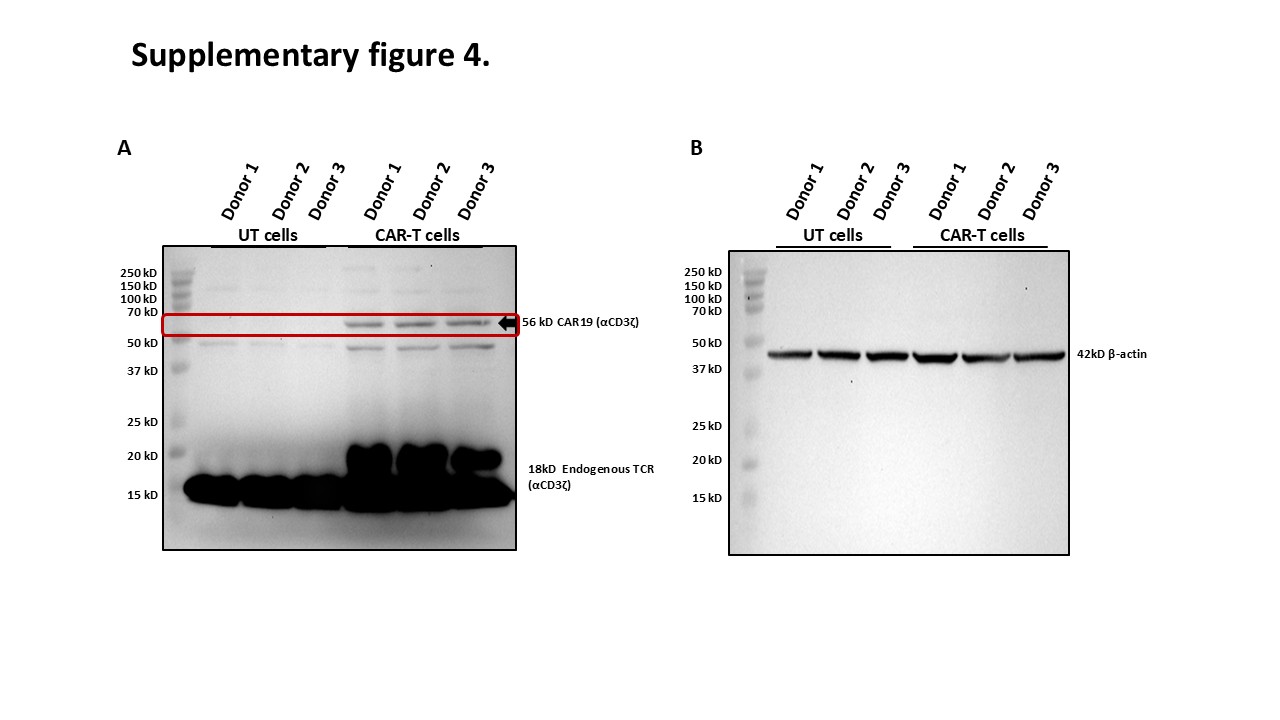

Supplement: Supplementary Figure 4 — Western blot analysis of CAR protein expression in primary T cells. The cell lysate was prepared from the primary T cells transduced with CAR and untransduced T cells from three different donors. The protein lysate was subjected to SDS-PAGE (12%) and immunoblotting with an (A) anti-CD3ζ antibody. β-Actin (B) was used as a loading control. The expected size of CAR is ~56 kDa. The protein ladder is mentioned on the left side of the blot. [file Image4.jpeg]

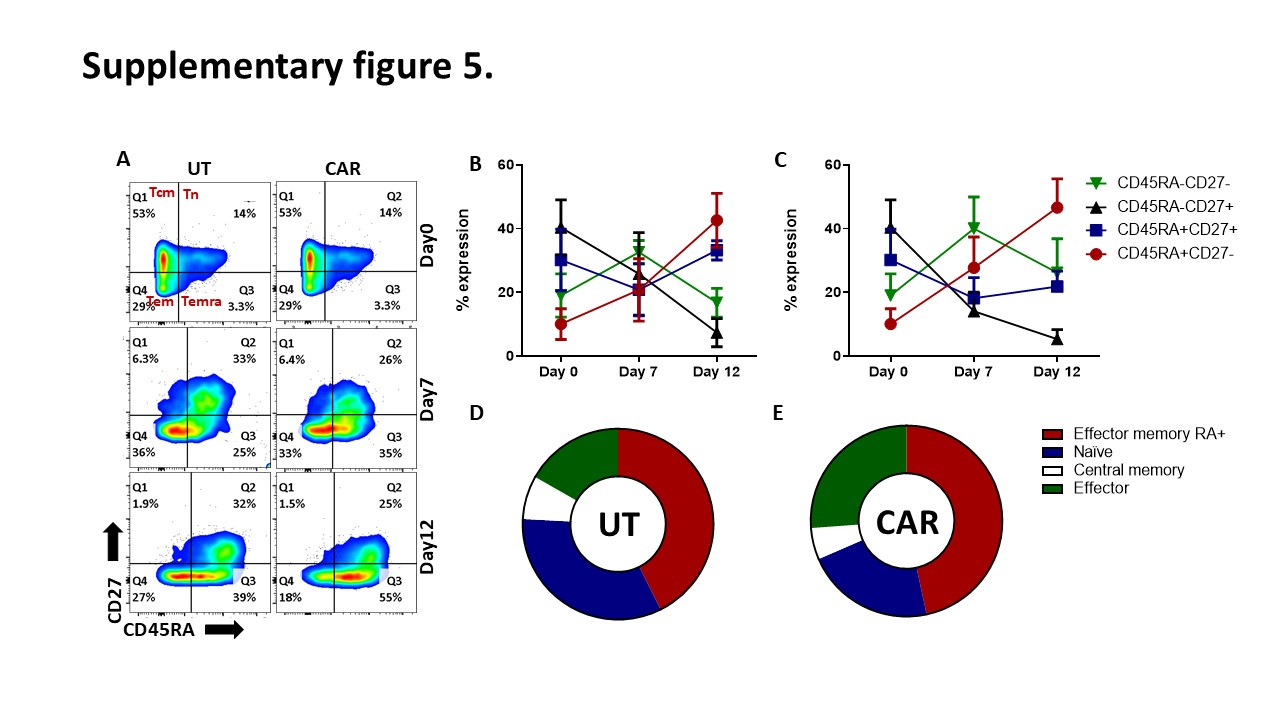

Supplement: Supplementary Figure 5 — Memory phenotype of CAR-T cells. CAR-T cells’ memory phenotype was measured using CD45RA and CD27 staining. (A) FACS plots representing untransduced and CAR transduced T cells for day 0, day 7 and day 12. (B, C) The graph represents the percentage expression of effector memory (CD45RA−CD27−), central memory (CD45RA−CD27+), naïve (CD45RA+CD27+), and RA+ effector memory (CD45RA+CD27−) subsets in untransduced (B) and CAR-transduced (C) T cells, measured across five different expansions (n=5). (D, E) Representative pie chart showing only day 12 of untransduced (D) and CAR transduced cell (E) expansions. Data is represented as mean ± SEM. [file Image5.jpeg]

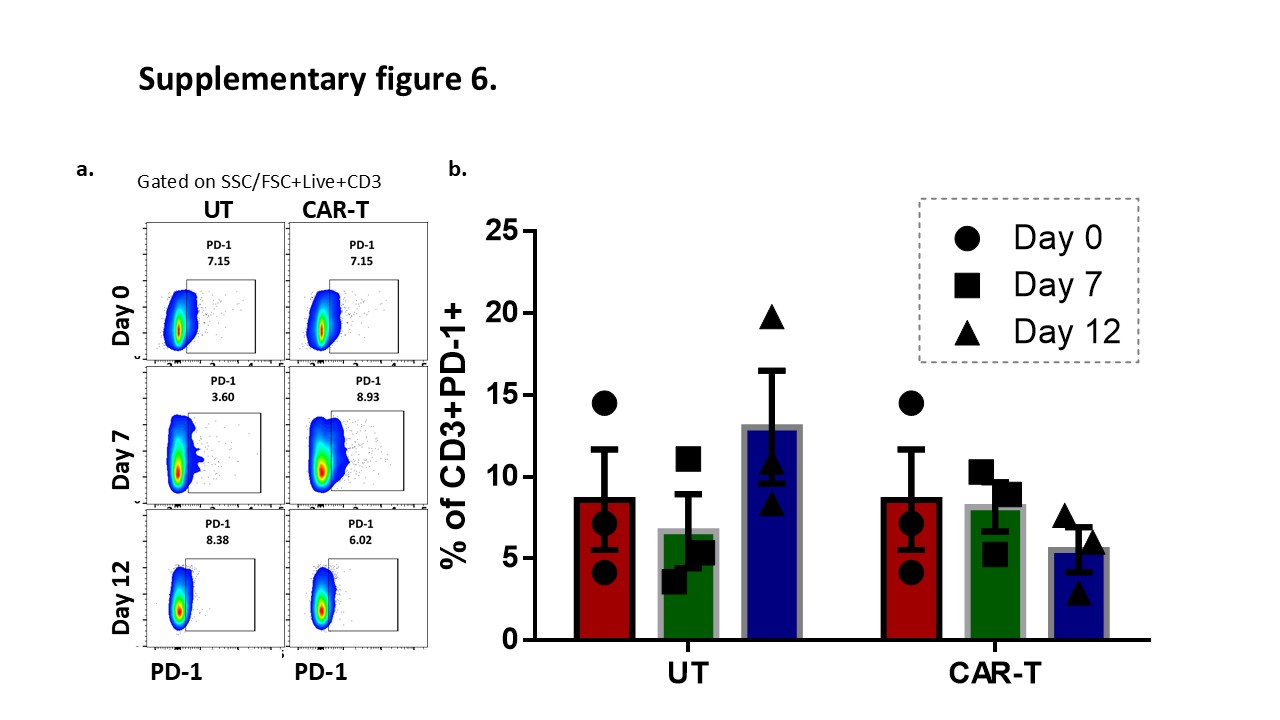

Supplement: Supplementary Figure 6 — PD-1 expression during CAR-T cell expansion. (A) Representing FACS plot of PD-1 expression measured on days 0, 7, and 12 during CAR-T cell expansion using a flow cytometer. (B) Data are represented as a bar graph of the percentage of PD-1 on untransduced T cells, and CAR-T. The data is represented as mean ± SEM (n=3). The statistical significance was estimated by using the Student’s T-test. There were no significant differences between UT and CAR-T cells during expansion. [file Image6.jpeg]

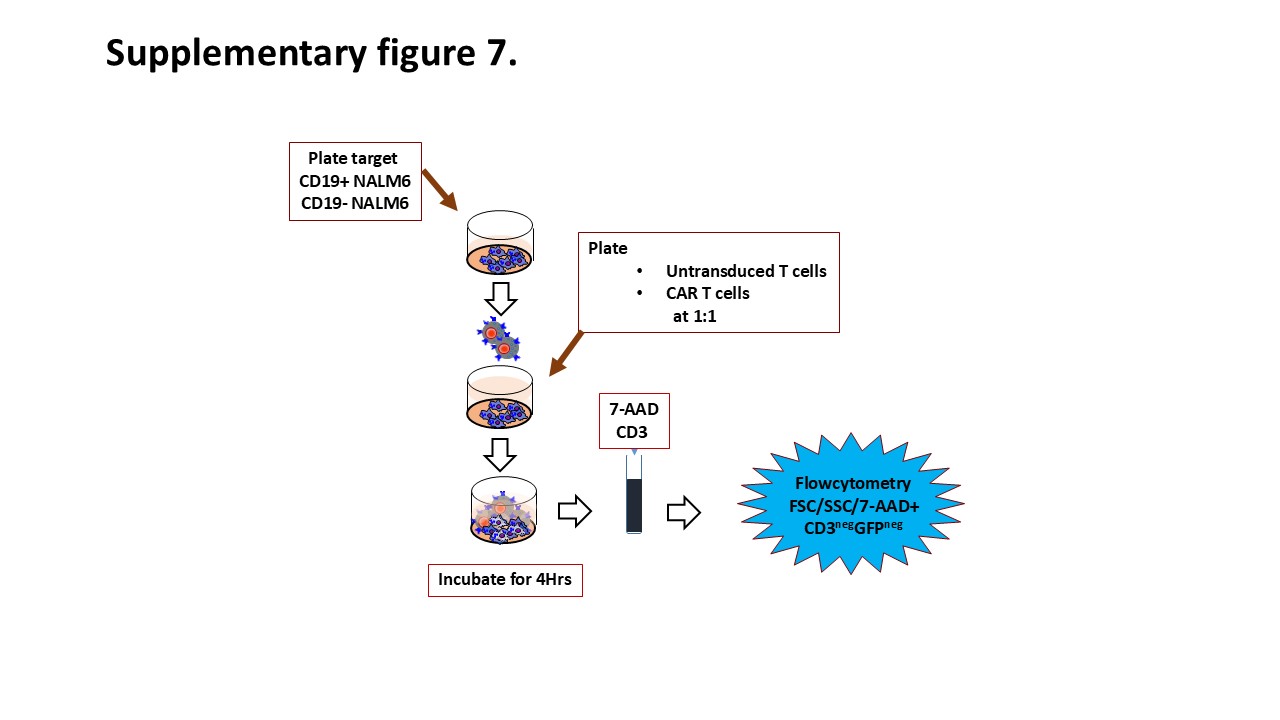

Supplement: Supplementary Figure 7 — Schematic representative of cytotoxicity assay. The Target(T) NALM-6 cells (CD19 + and CD19 -) were plated at 105 cells/well in a U bottom 96 cell plate in a serum-free media. The effector (E) cells (CAR-transduced cells) and untransduced controls were plated against the targets at a 1:1 (effector-to-target) ratio for 4 hours. The cells were stained with 7-AAD and CD3 antibody and acquired using flow cytometry. They were then serially gated on FSC/SSC gates to exclude the debris and 7-AAD to exclude the dead cells. GFP- CD3- cells were gated for measuring the survival of the target cells. [file Image7.jpeg]

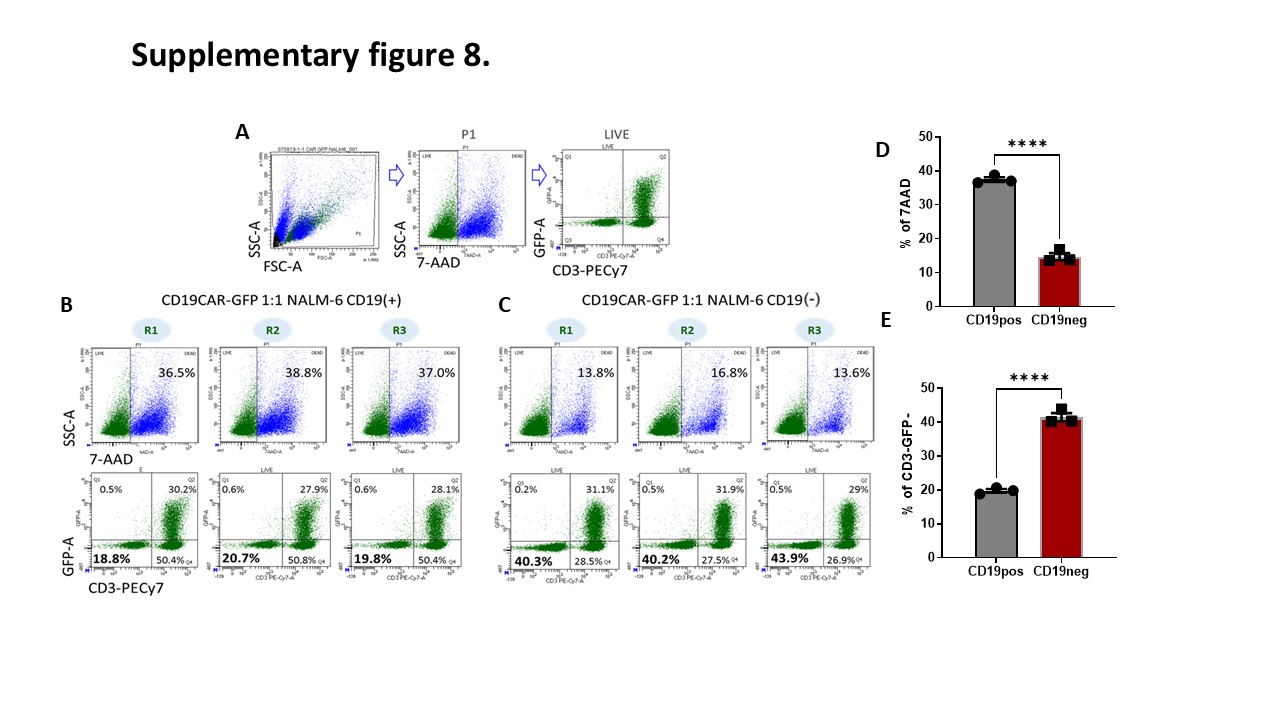

Supplement: Supplementary Figure 8 — Cytotoxicity of CD19 CAR against an equal number of WT and CD19KO NALM-6 cells. (A) Gating strategy to assess the cytotoxicity of CAR T cells against CD19 positive and CD19 negative Nalm-6 cells. The first gate excludes the debris, followed by gating at 7-AAD negative live population. GFP+ and CD3- cells are monitored to assess the survival of Nalm-6 cells. (B) FACS plots represent the survival of CD19-positive Nalm-6 cells when co-cultured with CAR T cells at a 1:1 ratio in triplicates. (C) FACS plots represent the survival of the CD19-negative Nalm-6 cells. The upper row represents cytotoxic cell lysis by 7-AAD staining alone. (D, E) The percentage of 7AAD (D) and survival of tumor cells (E) are depicted as mean ± SEM (r=3). The statistical significance is estimated by using the Student’s T-test. ****p<0.0001. [file Image8.jpeg]

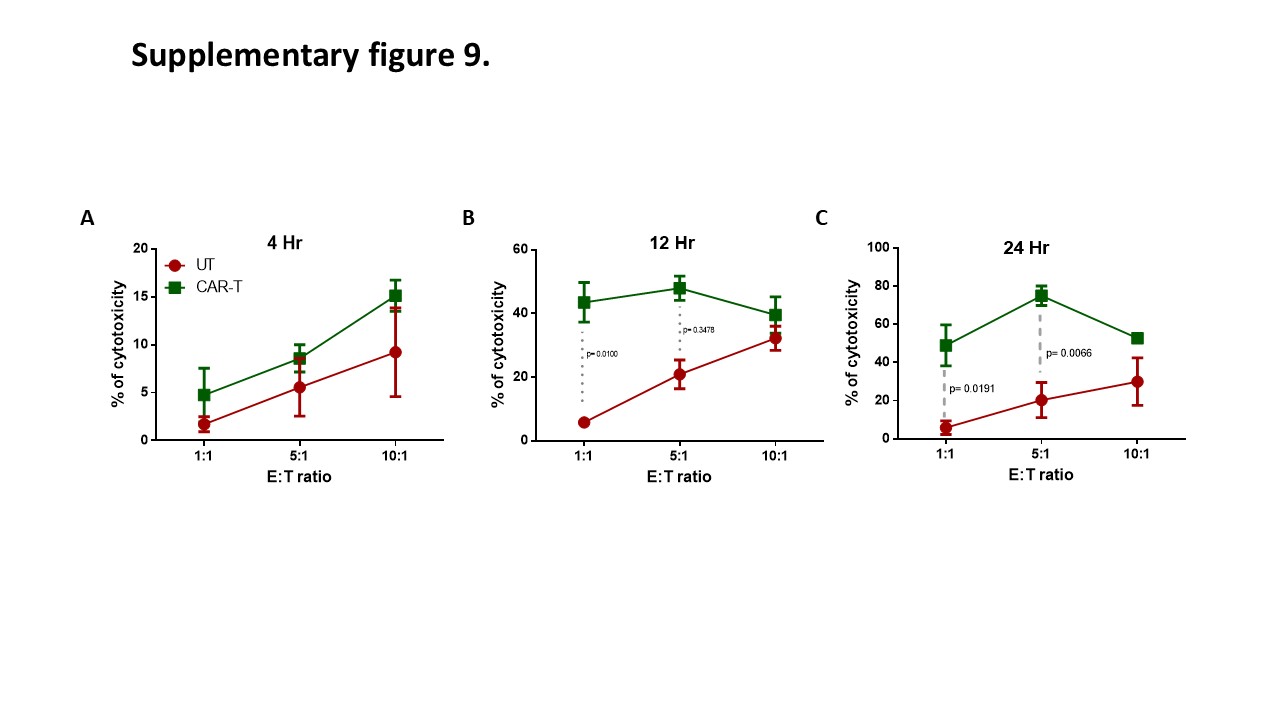

Supplement: Supplementary Figure 9 — Cytotoxicity of CAR-T cells at various E:T ratios and time points. CAR-T cells and untransduced T cells (effector) were co-cultured with nalm-6 cells (target) at different effector-to-target ratios (1:1, 5:1, and 10:1) at multiple time points (4 (A), 12 (B), and 24 hours (C)). Target cell lysis was measured using a flow cytometry-based cytotoxicity assay. The percentage of cytotoxicity is plotted against various E:T ratios. Data represent mean ± SEM from three different donors (n=3). The statistical significance was estimated by using the Student’s T-test. *p < 0.05, and **p<0.005. [file Image9.jpeg]

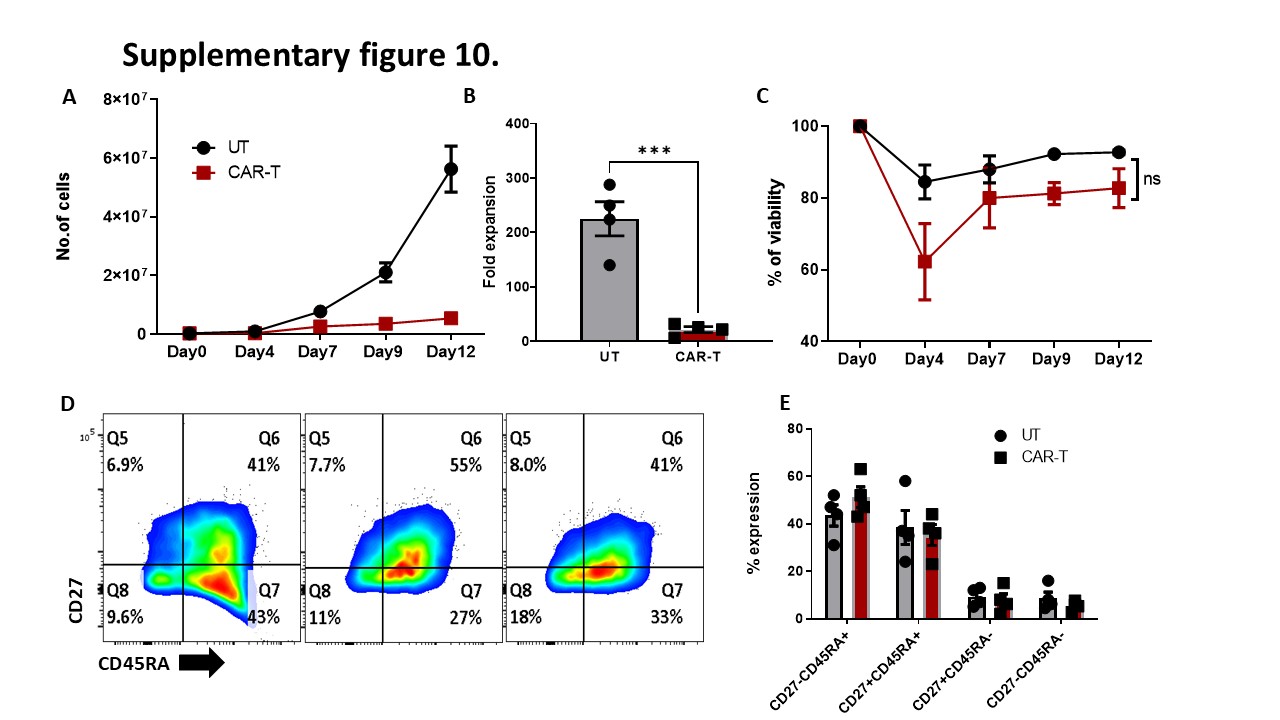

Supplement: Supplementary Figure 10 — Proliferation and differentiation of T cells transduced with cGMP grade CAR constructs. (A) The graph represents the number of untransduced and CAR-transduced T cells at the indicated days after initiating the T cell expansion culture. (B) Fold expansion of the untransduced and CAR-transduced T cells on day 12. (C) Viability of the untransduced and CAR-transduced T cells in the expansion culture. (D) Naïve (CD45RA+CD27+), T-cell effector RA positive (CD45RA+, CD27-), central memory (CD45RA-CD27+), effector memory (CD45-CD27-) profile of the CAR T cells on day 12. (E) The bar graph shows only day 12 of untransduced T cell and CAR-transduced T cell expansions. Data is represented as mean ± SEM (n=4). The statistical significance is estimated by using the Student’s T-test. ***p<0.0001, ns: not significant. [file Image10.jpeg]
